# Analyzing the impacts of cadmium alone and in co-existence with polypropylene microplastics on wheat growth

**DOI:** 10.3389/fpls.2023.1240472

**Published:** 2023-08-10

**Authors:** Zhiwei Han, Raheel Osman, Yi Liu, Zhangdong Wei, Lin Wang, Ming Xu

**Affiliations:** ^1^ Miami College, Henan University, Kaifeng, China; ^2^ College of Geography and Environmental Science, Henan University, Kaifeng, China; ^3^ Henan Key Laboratory of Earth System Observation and Modeling, Henan University, Kaifeng, China; ^4^ BNU-HKUST Laboratory for Green Innovation, Beijing Normal University, Zhuhai, China

**Keywords:** seed germination, seedling growth, heavy metal, particle size, enzyme activity

## Abstract

Heavy metals typically coexist with microplastics (MPs) in terrestrial ecosystems. Yet, little is known about how the co-existence of heavy metals and MPs affect crops. Therefore, this study aimed to evaluate the impact of cadmium (Cd; 40 mg/L) alone and its co-existence with polypropylene (PP)-MPs (50 and 100 µm) on seed germination, root and shoot growth, seedling dry weight (DW), and antioxidant enzyme activities of wheat. The study demonstrated that the germination rate of wheat did not vary significantly across treatment groups. Yet, the inhibitory impact on wheat seed germination was strengthened under the co-existence of Cd and PP-MPs, as the effect of a single treatment on seed germination was non-significant. The germination index and mean germination time of wheat seeds were not affected by single or combined toxicity of Cd and PP-MPs. In contrast, Cd and PP-MPs showed synergistic effects on germination energy. Wheat root and shoot length were impeded by Cd alone and in combination with PP-MPs treatments. The DW of wheat seedlings showed significant change across treatment groups until the third day, but on the seventh day, marginal differences were observed. For example, on third day, the DW of the Cd treatment group increased by 6.9% compared to CK, whereas the DW of the 100 µm PP-MPs+Cd treatment group decreased by 8.4% compared to CK. The co-occurrence of Cd and PP-MPs indicated that 50 μm PP-MPs+Cd had an antagonistic impact on wheat seedling growth, whereas 100 μm PP-MPs+Cd had a synergistic impact due to the larger size of PP-MPs. The antioxidant enzyme system of wheat seeds and seedlings increased under single Cd pollution, while the activities of superoxide dismutase, catalase, and peroxidase were decreased under combined pollution. Our study found that Cd adversely affects wheat germination and growth, while the co-existence of Cd and PP-MPs have antagonistic and synergistic effects depending on the size of the PP-MPs.

## 1 Introduction

The term “plastic” derives from the Latin word “plasticus,” which is emanated from the Greek word “plastikos,” meaning something that could be molded or suitable for molding (Plastics Europe, 2018). The plastic pollution issue emerged in 1950 with the onset of commercial manufacturing (Geyer et al., 2017). However, this large-scale production and consumption steadily increase the amount of plastic in our surroundings. Global plastic production rose ~12% in 2021 (compared to production in 2017) to more than 390 million tons, with China reaching almost one-third (32%) of global plastic production (Plastics Europe, 2022).

Plastics pose a serious challenge, as they have limited biodegradability, resulting in their accumulation rather than decomposition when released into the environment or dumped in landfills (Geyer et al., 2017). The term microplastics (MPs) are referred to various plastic particles having diameters ≤ 5 mm (Rummel et al., 2017; Iqbal et al., 2023). They pose a new planetary threat due to their characteristics of refractory degradation and easy migration and are found in air, water, soil, and other environmental media (Eerkes-Medrano et al., 2015; Rummel et al., 2017). The MPs are formed when plastic is transformed into small debris through pyrolysis, ultraviolet radiation, aging, and biodegradation (Qi et al., 2018). Additionally, owing to their large surface area and high hydrophobicity, MPs’ surfaces easily absorb contaminants from their surrounding environment (Rummel et al., 2017). Therefore, the pollution and ecotoxicological effects of MPs need to be further studied. Initially, most investigations focused on characterizing and quantifying MPs in marine ecosystems (Yu et al., 2020b), although there are relatively few studies that investigated environmental behavior and impact mechanism of MPs in agroecosystems, which are considered to be the most MP-contaminated terrestrial system (Boots et al., 2019; de Souza Machado et al., 2019; Sun et al., 2020; Fan et al., 2022). In agroecosystems, the primary sources of MPs include plastic mulching, sewage irrigation, and solid waste (sludge application) (Nizzetto et al., 2016; van den Berg et al., 2020). The MPs entering the soil ecological environment system not only affect the physiochemical attributes and functions of the soil but also have deleterious effects on the growth of animals and plants, community structure, and microbial diversity in the soil (Boots et al., 2019; Zhou et al., 2021b).

Previous studies that investigated the impacts of MPs on plant growth have mainly focused on wheat (Taylor et al., 2020; Gong et al., 2021; Liu et al., 2021; Pflugmacher et al., 2021; Qi et al., 2018; Lian et al., 2020a; Lian et al., 2020b; Gu et al., 2021; Zong et al., 2021), mung bean, soybean (Wang et al., 2021b), spring onion (Maity et al., 2020; de Souza Machado et al., 2019), and rice (Dong et al., 2022; Kaur et al., 2022). Qi et al. (2018) conducted a pot experiment with micro- and macro-plastics and noted that starch-based biodegradable plastic film possessed a higher deleterious effect on wheat growth than low-density polyethylene. A study by Bosker et al. (2019) demonstrated that the germination rate of water celery was significantly reduced by MPs of various sizes (50, 500, and 4,800 nm) due to their accumulation on seed case, and the negative impact augmented with plastic size. Moreover, Boots et al. (2019) evaluated the biophysical response of MPs (high-density polyethylene and biodegradable polylactic acid) on the growth of earthworms and ryegrass in soil. They discovered that MPs affected plant production, decreased earthworm biomass, and altered the soil properties. Yet, there are disparities about the impacts of MPs of different sizes on plant development and their contagiousness to plants. For example, MPs (particularly polyvinylchloride) have been reported to inhibit mineral bioaccumulation through the rhizosphere and alter plant growth and development due to oxidative burst and increase in hydrogen peroxide, aminolaevulinic acid, and proline concentrations (Pignattelli et al., 2020). Furthermore, de Souza Machado et al. (2019) reported negligible impacts of polyester terephthalate, PP-MPs, and polyethylene-MPs in spring onion; however, polystyrene-MPs depict the increase in root biomass with polyethylene terephthalate-MPs demonstrated a decrease in stem biomass. These discrepancies among studies could be due to the MPs concentration, material, and particle size (de Souza Machado et al., 2019; Kaur et al., 2022; Xu et al., 2022).

Besides MPs pollution, agricultural lands are also polluted by heavy metals (Abbasi et al., 2020; Wang et al., 2021c; Kaur et al., 2022), with cadmium pollution (Wang et al., 2021a) being particularly prominent (Haider et al., 2022; Kaur et al., 2022). These heavy metals accumulate in the soil, causing harm to the soil and the growth of soil microbial communities and plants (Abbasi et al., 2020; Wang et al., 2020b; Haider et al., 2022). The heavy metals availability and toxicity in plants are affected by MPs through adsorption, aggregation, bioaccumulation, and chelation (de Souza Machado et al., 2019; Kaur et al., 2022; Wen et al., 2022; Iqbal et al., 2023). Past investigations demonstrated that MPs could adsorb heavy metals and flow up the food chain, ultimately threatening human health (Abbasi et al., 2020; Kaur et al., 2022; Iqbal et al., 2023; Khan et al., 2023). The MPs can form complexes with heavy metals by chelating them, which may increase metal solubility and plant uptake (Wen et al., 2022). Moreover, MPs alter the availability of heavy metals by changing soil aggregation, bulk density, and soil redox status. The MPs with high adsorption capacity, hydrophobicity, and surface area can absorb heavy metals (Gao et al., 2021; Wang et al., 2019), changing their bioavailability and mobility (Yu et al., 2020a). Yet, the effects of MPs on heavy metals on bioaccumulation and toxicity in wheat vary across studies. The MPs may abate or exacerbate heavy metal availability and absorption by plants. For example, polystyrene-MPs were reported to hinder the toxicity and accumulation of copper and Cd in wheat seedlings (Zong et al., 2021). In contrast, Lian et al. (2020b) discovered a marginal reduction in Cd toxicity in the presence of polystyrene-MPs. Previously, it has been proven that heavy metals increase reactive oxygen species (ROS) and impede chlorophyll content, illustrating their deleterious effects on photosynthesis and antioxidant system, leading to alteration in plant anatomy and morphology (Manzoor et al., 2022; Khan et al., 2023). Plant antioxidant systems defend plants from heavy metal stress-induced oxidative stress and ROS (Manzoor et al., 2022; Khan et al., 2023). Heavy metals and MPs may interact, affecting heavy metal availability and toxicity (Yu et al., 2020a; Gu et al., 2021; Zong et al., 2021; Kaur et al., 2022).

Wheat, as one of the most extensively consumed crops, is critical to global food security (Osman et al., 2022), providing 20% of daily calories and proteins for 4.5 billion people (Shewry and Hey, 2015). The MPs and heavy metals adversely influence the growth and development of wheat (Lian et al., 2020b; Zong et al., 2021). The Cd toxicity and tolerance levels may differ depending on the genotype and growth stage. In particular, the germination process of plants is a critical stage of their growth cycle that is highly responsive to environmental factors and toxicity (Kaur et al., 2022; Iqbal et al., 2023).

Furthermore, MPs can affect heavy metal toxicity and accumulation in wheat. However, to best of our knowledge, no attempt has been made to assess the combined impact of Cd and polypropylene (PP)-MPs on wheat seed germination and seedling growth, which could threaten the environmental sustainability, food security, and human health. Therefore, it is crucial to assess the effects of Cd alone and in combination with PP-MPs on seed germination and seedling growth of wheat, which serves as a significant indicator of toxicity. Hence, this study aims to quantify the impact of Cd on wheat germination, seedling growth, and enzyme activities alone and in combination with PP-MPs of different particle sizes (50 and 100μm).

## 2 Materials and methods

### 2.1 Preparation of microplastic suspension and cadmium solution

The current study employed two different sizes of PP-MPs: 50 and 100 μm. The PP-MP (purchased from BaseLine Chromtech Research Centre in Tianjin, China) was chosen for this study due to its pervasiveness in the soil ecosystem, while 50 and 100 μm PP-MPs were selected to assess the impact of the size of PP-MP on wheat seedlings. The concentration of the PP-MPs suspension was set to 500 mg/L to facilitate the interaction between PP-MPs and wheat seedlings, following Wang et al. (2021b) and Shi et al. (2022). Prior to the germination test, the development of aggregates in the MP solution was minimized by 1.5 h of ultrasonic treatment (25°C, 40 kHz). After sonicating, the MP solution was uniformly disseminated in the liquid phase and retained in beakers for subsequent use. The 40 mg/L cadmium (Cd^2+^) solution (Xia et al., 2018) was made by using a high-quality cadmium nitrate tetra-hydrate salt (Kaur et al., 2022).

### 2.2 Seed germination test

The widely grown winter wheat cultivar Yumai 49-198 was used to conduct experiments. The germination test was conducted with minimal modifications to the protocol explained by Wang et al. (2021b). The wheat seeds were sterilized with sodium hypochlorite solution (2% (v/v)) for 30 min to inhibit microbial contagion and subsequently washed with demineralized water to erase residual solution. Afterward, 10 healthy seeds of the same size were placed in a 90-mm Petri dish lined with two layers of Whatman No. 1 filter paper. The four treatments were replicated six times, *viz*, CK, Cd-40 mg/L, 50 μm PP-MPs+Cd, and 100 μm PP-MPs+Cd in Petri dishes and placed in a growth incubator at 25°C and 60% relative humidity with a 12-h diurnal cycle. Daily at 8 a.m., the number of germinated seeds (germination was considered successful once the root length surpassed half the seed length) were counted. After each measurement, 2 ml of distilled water was supplemented to Petri dishes utilizing a pipette gun to avoid water stress. Sampling was done on the third and seventh day for each treatment by measuring root and shoot length followed by oven drying at 105°C for 24 h to constant mass to determine the DW. To investigate the synergistic or antagonistic impacts of Cd alone and in conjunction with PP-MPs on wheat seed germination and seedling growth, measurements were made for seed germination rate (GR), germination index (GI), germination vigor (GV), germination energy (GE), and mean germination time (MGT), along with root and shoot length and weight. The GR is defined as the average number of seeds germinating over a specific period. The GI represents the number of seeds successfully germinated each day to the total number of days. Yet, GV defines the seed germination rate within a given period, and GE is the proportion of viable seeds in a given sample that can germinate under favorable conditions. **Table 1** displays the calculation formulas for the seed vigor indices.

**Table 1:** Calculation formulas of seed vigor indices.

Seed vigor Indices	Formulas
Germination Rate (GR, %)	$\text{GR }=\left(\frac{N_{7d}}{N_{t}}\right)\times 100\%$
Germination Index (GI)	$\text{GI}=\sum^{}\frac{G_{i}}{D_{i}}$
Mean Germination Time (MGT, d)	$\text{MGT}=\frac{\sum^{}\left(D_{i}\times G_{i}\right)}{\sum^{}G_{i}}$
Germination Energy (GE, %)	$\text{GE}=\frac{N_{3d}}{N_{t}}\times 100\%$

where N_t_ represents the total number of seeds examined, N_3d_ and N_7d_ refers to the number of seeds that germinated on third and seventh day, respectively. D_i_ depicts the ith day of germination, G_i_ denotes the total number seeds germinated on D_i_, and d shows the number of days.

### 2.3 Enzyme activity

A set of enzyme activity kits obtained from Solarbio Company China was employed to test the activity of peroxidase (POD; Cat. No. BC00390), catalase (CAT; Cat. No. BC00200), and superoxide dismutase (SOD; Cat. No. BC0170) in wheat seedlings on the seventh day of the experiment. The CAT, POD, and SOD enzyme activities were determined using formulas mentioned in the kit. The final supernatant was collected after centrifuging 100 mg of blended plant tissue at 8,000 rpm for 10 min at 4°C to determine enzyme activity.

#### 2.3.1 Peroxidase activity

For estimation of POD activity, 100 mg of tissue was added in 1 mL of extract for ice bath homogenization, succeeded by centrifugation at 8,000 rpm for 10 min. Next, add 15 µL of the supernatant to a 1-mL glass cuvette. Then, sequentially add 270 µL of distilled water, 520 µL of Reagent-1, 130 µL of Reagent-2, and 135 µL of Reagent-3 to the cuvette. Afterward, the absorbance at 470 nm was assessed using a UV-3600 spectrophotometer (Shimadzu, Kumamoto, Japan). After 30 min, the initial absorbance (A1) was determined, and after 1 min, the final absorbance (A2) was recorded. The change in absorbance was ascertained by calculating the difference between A2 and A1. The Δ470 value represented a change in enzyme activity of 0.01 units per minute per gram of tissue in a per milliliter reaction system. The POD activity was estimated as follows:


POD (U/g)=7133× Δ A÷ W

where W is the sample mass in grams.

#### 2.3.2 Catalase activity

The CAT activity was determined by weighing 100 mg of plant tissue and adding 1 mL of extract for ice bath homogenization and then centrifuging at 8,000 rpm for 10 min at 4°C. This was followed by the preparation of detection sample solution having 50 μL Reagent-2 plus 13 μL Reagent-1, which were mixed thoroughly and put in the water bath for 10 min at 25°C. Then, 1 mL of detection solution and 35 μL of supernatant were combined and mixed well for 5 s in a quartz colorimetric dish. The initial absorbance (A1) was noted after 30 s at 240 nm, and the second absorbance (A2) was recorded after 1 min. The ΔA was calculated by subtracting A2 from A1. The CAT activity was defined as catalytic degradation of 1 μmol H_2_O_2_ per g of tissue per minute in the reaction system. The CAT activity was estimated as follows:


CAT (U/g)=764.5×ΔA÷W

where W is the sample mass in grams.

#### 2.3.3 Superoxide dismutase activity

To determine SOD activity, 1 mL of the extract was employed for ice bath homogenization along with 100 mg of plant tissue. The mixture was then centrifuged at 8,000 rpm at 4°C for 10 min. After that, a sample tube was prepared containing 90 μL of supernatant, 240 μL of Reagent-1, 6 μL of Reagent-2, 180 μL of Reagent-3, 480 μL of distilled water, and 30 μL of Reagent-5. After that, the control tube was also prepared, which contained 90 μL of supernatant, 240 μL of Reagent-1, 180 μL of Reagent-3, 486 μL of distilled water, and 30 μL of Reagent-5.

Two blank tubes were prepared as well. Blank tube-1 consisted of 240 μL of Reagent-1, 6 μL of Reagent-2, 80 μL of Reagent-3, 570 μL of distilled water, and 30 μL of Reagent-5. While Blank tube-2 had 240 μL of Reagent-1, 180 μL of Reagent-3, 576 μL of distilled water, and 30 μL of Reagent-5. Then, each test tube solution was thoroughly mixed and placed in a 37°C water bath for 30 min. Following incubation, the absorbance at 560 nm was determined using a 1-mL glass colorimeter. The recorded absorbance value was as follows: A1 A-test, A-control, A1-blank, and A2-blank.

The following calculations were performed to calculate the SOD activity: A-test =A-test – A-control, A-blank =A1-blank − A2-blank, inhibition percentage = (A-blank − A-test) × 100%.

Finally, the SOD activity was calculated using the formulas: A-control, A1-blank, and A2-blank, respectively, calculate △A-measure =A-measure − A-control, △A-blank =A1-blank − A2-blank, and inhibition percentage = (△A-blank −△A-measure) ÷△A-blank ×100%:


SOD (U/g)=11.4×percent of inhibition÷(1- percent of inhibition)÷W×F

where W is the sample mass in grams, and F is the sample dilution ratio.

### 2.4 Data analysis

The data for each concentration were processed in R version 4.2.3 using the dplyr package (version 1.1.2) to determine the mean ± standard deviation. Further analysis of variance (ANOVA) was performed by using the agricolae version 1.3-5 package, and multiple *post-hoc* tests were performed by applying Tukey Honestly significant difference (HSD) test. The graphs (bar plots) were generated employing the ggplot2 (version 3.4.2).

## 3 Results

### 3.1 Effects of Cd and PP-MPs on seed germination rate and seed vigor index

The seed GR did not differ significantly among various treatment groups (**Table 2**, **Supplementary Figure S1**). In particular, the Cd (40 mg/L) treatment group showed no difference in seed GR compared to the CK treatment group. However, the GR in the two groups supplemented with PP-MPs (500 mg/L, 50 and 100 µm) showed an enhanced inhibitory effect on wheat seed GR (**Supplementary Figure S1**). For example, in our study, the decrease in GR for 50 µm PP-MPs was 4.3%, while it was 26% for 100 µm PP-MPs.

**Table 2:** Seed vigor indices of wheat seeds exposed to PP and Cd at different concentrations.

Treatment	Germination Rate (GR, %)	Germination Index (GI, 3d)	Germination Index (GI, 7d)	Mean Germination Time (MGT, 3d)	Mean Germination Time (MGT, 7d)	Germination Energy (GE, %)
CK	76.7 ± 20.8a	4.89 ± 0.77ab	10.6 ± 0.89ab	2.56 ± 0.04b	4.68 ± 0.13a	66.7 ± 5.8a
Cd	76.7 ± 5.8a	5.94 ± 1.64a	11.7 ± 3.25a	2.51 ± 0.02b	4.55 ± 0.06a	73.3 ± 23.1a
50 µm PP-MPs+Cd	73.3 ± 11.5a	3.56 ± 0.67ab	8.3 ± 1.1ab	2.54 ± 0.04b	4.81 ± 0.18a	46.7 ± 5.8a
100 µm PP-MPs+Cd	56.7 ± 5.8a	2.83 ± 0.44b	6.97 ± 0.58b	2.68 ± 0.06a	4.83 ± 0.16a	50 ± 10a
*p*-level	ns	*	*	**	ns	ns

Different alphabets represent the significant differences between treatments groups. CK means control check, Cd means cadmium (40 mg/L), and PP means polypropylene. p-level is given; *p< 0.05; **p< 0.01; ns, non-significant.

Regarding seed GI, Cd treatment demonstrated a non-significant increase in the seed GI on the third (21.6%) and seventh (10.6%) day compared to the CK group (**Table 2**, **Supplementary Figure S2**). However, the addition of PP-MPs in the Cd experimental group inhibited the seed GI on both sampling days (third and seventh day). Notably, the large size PP-MPs (100 μm) and Cd significantly (p< 0.05) reduced the GI of wheat seedlings compared to CK. The GI was reduced by 27.3% and 21.4% for 50 µm PP-MPs+Cd on the third and seventh day, while GI was reduced by 42% and 32% under 100 µm PP-MPs+Cd on the third and seventh day, respectively. In the case of MGT, on the third day of the experiment, there was no substantial change among CK, Cd, and 50 µm PP-MPs+Cd, while 100 µm PP-MPs+Cd showed a substantial increase (4.8% compared to CK), suggesting that PP-MPs-MPs with higher particle size significantly manipulate the MGT in wheat seedlings. However, on the seventh day, the MGT of the Cd treatment group reduced by 2.7% compared with CK, while 50 and 100 µm PP-MPs+Cd depicts increase in MGT by 2.8% and 3.3% (**Table 2**, **Supplementary Figure S3**). In terms of GE, treatment with 40 mg/L Cd increased the GE of wheat seeds by 10% compared to CK, as demonstrated in **Table 2** and **Supplementary Figure S4** (p > 0.05). The MPs in combination with Cd (50 µm PP-MPs+Cd, 100 µm PP-MPs+Cd) reduced the GE of wheat seeds by 30% and 25%, respectively.

### 3.2 Effects of Cd and PP-MPs on root length and shoot length

**Figure 1:**
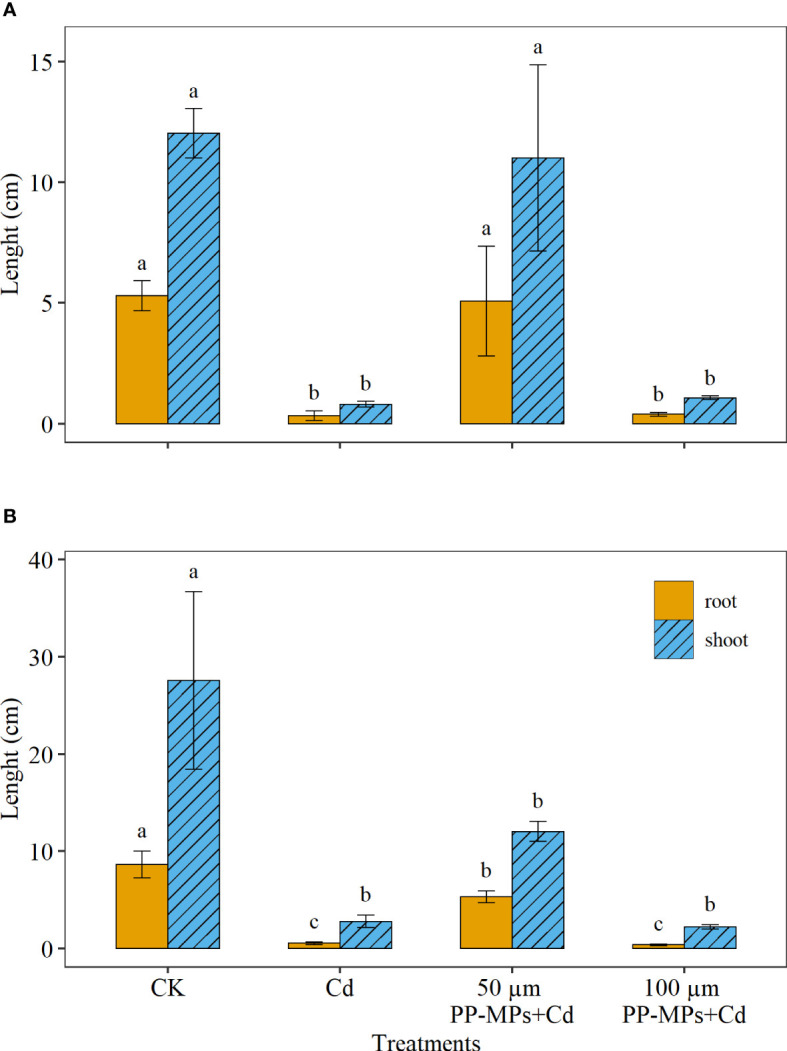
Impact of Cd (40 mg/L) and PP-MPs (50 and 100 µm) on the root and shoot length on the third day **(A)** and seventh day **(B)**. Values are the mean of six replicates ± standard deviation. Different alphabets represent the significant differences between treatment groups for root and shoot, where CK represents the control check, Cd represents the cadmium, and PP-MPs represents the polypropylene microplastics.

**Figure 1** depicts the significant differences between various treatments on wheat seedling growth and development. Compared with CK, all experimental groups showed a decrease in shoot and root length of wheat seedlings on the third and seventh day. Yet, the 50 µm PP-MPs+Cd treatment group showed a slight increase in root length on the seventh day, while shoot length showed a significant reduction compared to CK. The Cd treatment group and 100 µm PP-MPs+Cd treatment group showed strong inhibition for root and shoot length (**Figure 1**). For instance, root and shoot length reduced by approximately 92% for Cd and 100 µm PP-MPs+Cd treatment group compared with CK on both sampling dates.

### 3.3 Effects of Cd and PP-MPs on seedling dry weight

**Figure 2:**
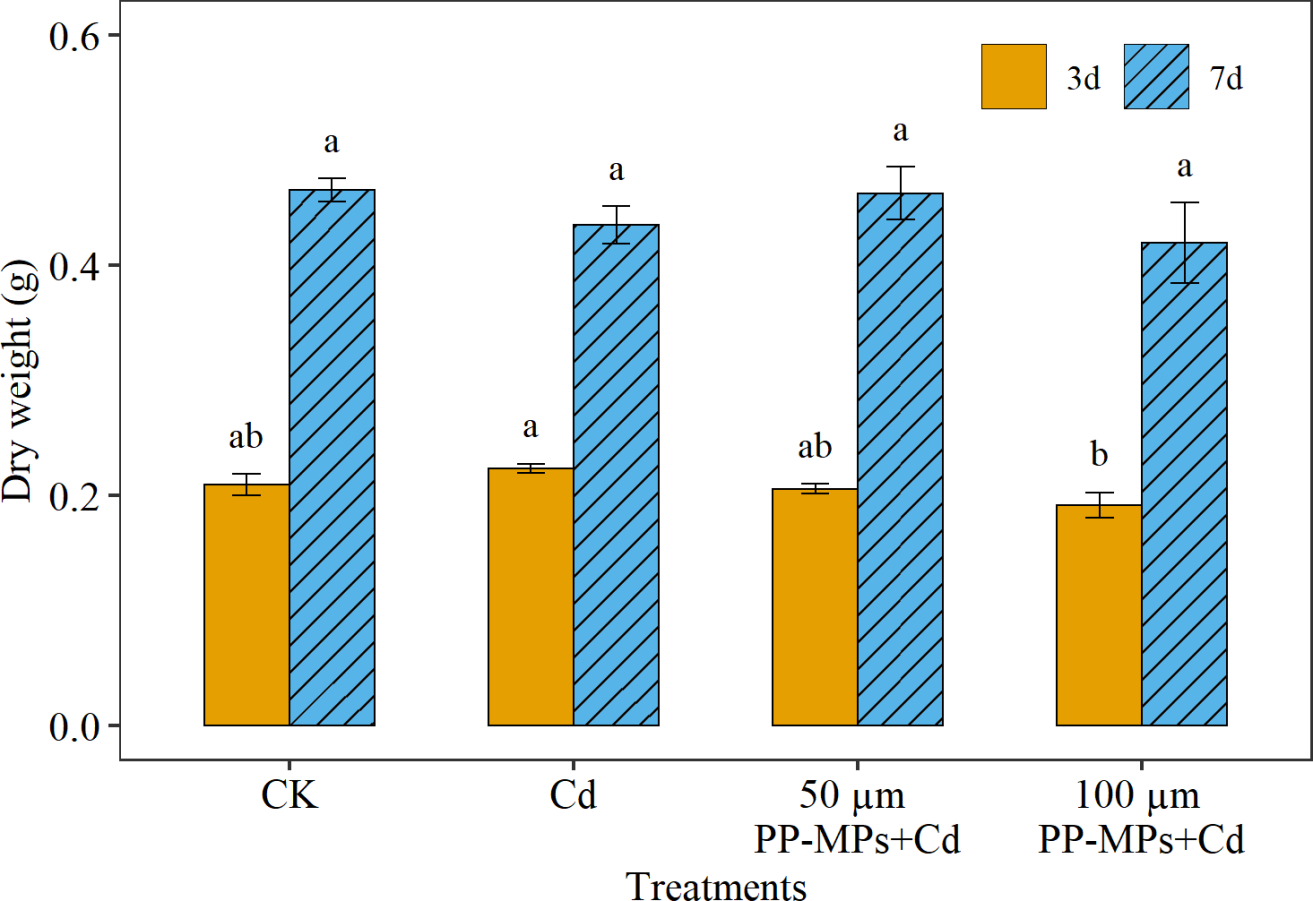
Impact of Cd (40 mg/L) and PP-MPs (50 and 100 µm) on dry weight on the third day and seventh day. Values are the mean of six replicates ± standard deviation. Different alphabets represent the significant differences between treatment groups on the third and seventh day, where CK represents the control check, Cd represents the cadmium, and PP-MPs represents the polypropylene microplastics.

On the third day of the experiment, the DW of wheat seedlings in all treatment groups differed significantly (**Figure 2**). The Cd treatment group showed a significant increase of approximately 6.9% in DW compared to the CK. In contrast, the 100 µm PP-MPs+Cd treatment group showed a significant reduction, with DW dropping to approximately 8.4% compared to CK. Yet, by the seventh day, no significant differences were observed between the various treatments groups and CK treatment group (**Figure 2**). The CK treatment group showed maximum DW of wheat seedlings on the seventh day, followed by 50 µm PP-MPs+Cd and Cd treatment groups. Interestingly, the100 µm PP-MPs+Cd treatment group depicted the maximum decline (9.9%) in seedling DW.

### 3.4 Effects of Cd and PP-MPs on the POD, CAT, and SOD activity

**Figure 3:**
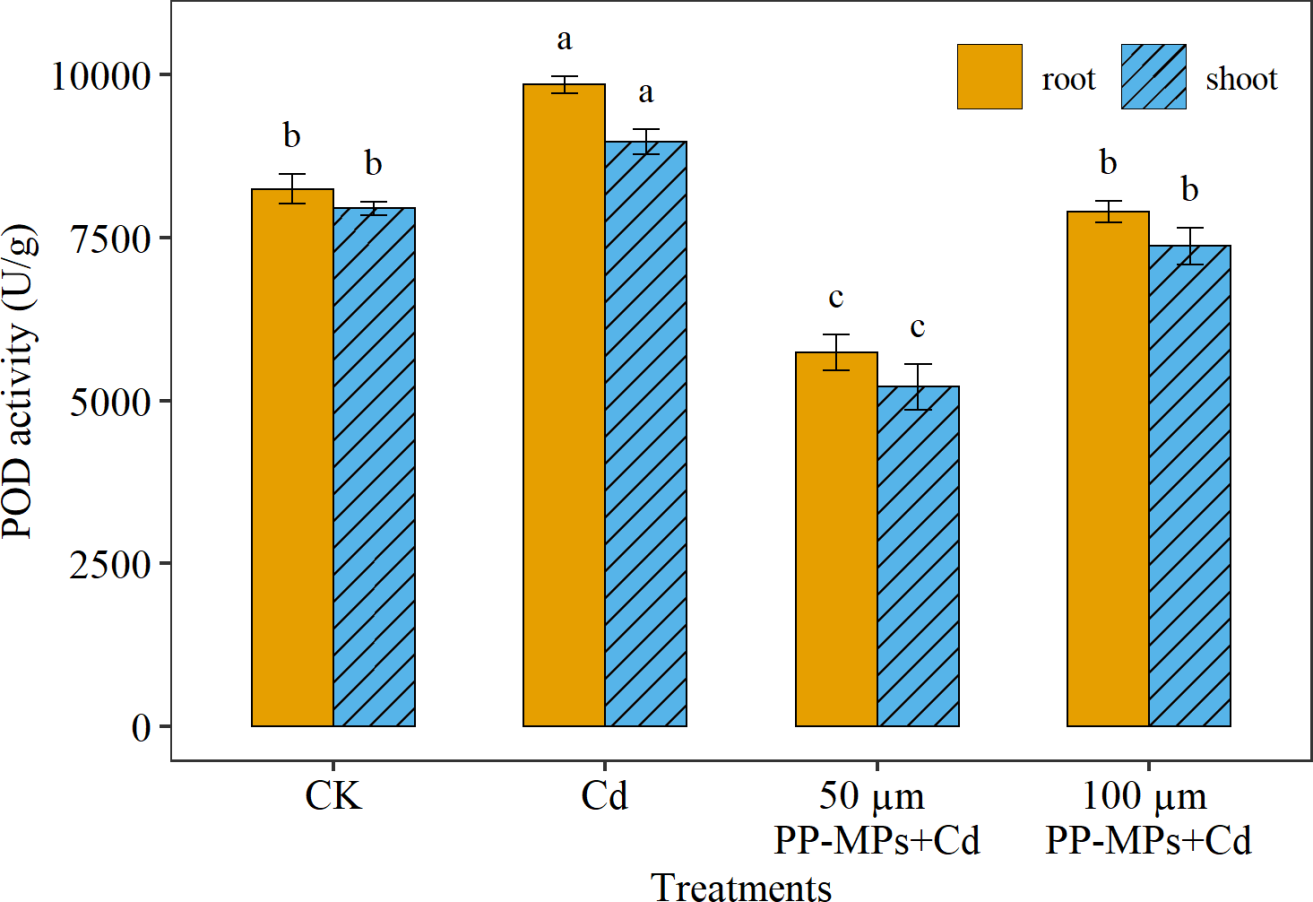
Impact of Cd (40 mg/L) and PP-MPs (50 and 100 µm) on peroxidase activity (POD) of the root and shoot on the seventh day. Values are the mean of six replicates ± standard deviation. Different alphabets represent the substantial differences between treatment groups for the root and shoot; where CK represents the control check, Cd represents the cadmium, and PP-MPs represents the polypropylene microplastics.

Among the root and shoot, the highest POD activity was recorded in the root (7% higher) compared to the shoot. Regarding various treatment groups, the Cd treatment group depicted a considerable increase (19%) in the POD activity of wheat seedlings relative to CK (in both root and shoot; **Figure 3**). However, with the addition of PP-MPs, the POD activity decreased significantly. The impact of various particle sizes of PP-MPs depicts considerable variation in POD activity in both root and shoot, with the impact of 50 μm PP-MPs+Cd treatment (30% decrease) being more pronounced than that of 100 μm PP-MPs+Cd treatment (4% decrease). A significant (p< 0.05) increase in POD enzyme activity was observed in the Cd treatment group (for both root and shoot). However, POD activity decreased significantly in the 50 µm PP-MPs+Cd treatment group.

**Figure 4:**
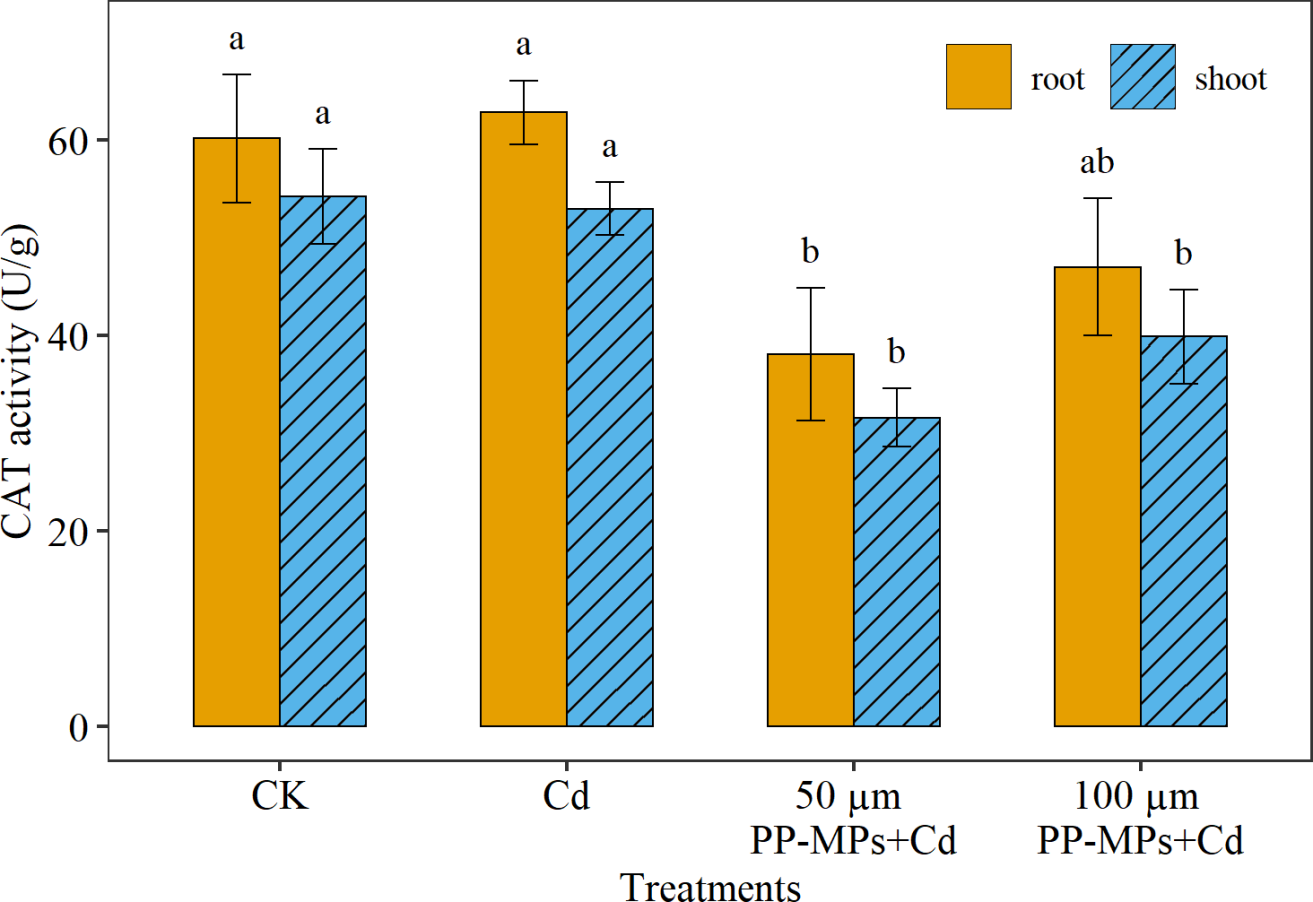
Impact of Cd (40 mg/L) and PP-MPs (50 and 100 µm) on catalase activity (CAT) of the root and shoot on the seventh day. Values are the mean of six replicates ± standard deviation. Different alphabets represent the substantial differences between treatment groups for the root and shoot, where CK represents the control check, Cd represents the cadmium, and PP-MPs represents the polypropylene microplastics.

Similar to POD activity, the highest CAT activity was recorded in the root (14% higher) than the shoot. Among various treatments, CAT activity did not show considerable change in the presence of Cd alone (p > 0.05; **Figure 4**). However, when Cd was combined with PP-MPs, the CAT activity reduced substantially. Yet, CAT activity did not show a significant discrepancy between particle sizes of PP-MPs. The 50 μm PP-MPs+Cd treatment reduces CAT activity in root and shoot by 36% and 42%, respectively, while the 100 μm treatment decreases it by 21% and 26%.

**Figure 5:**
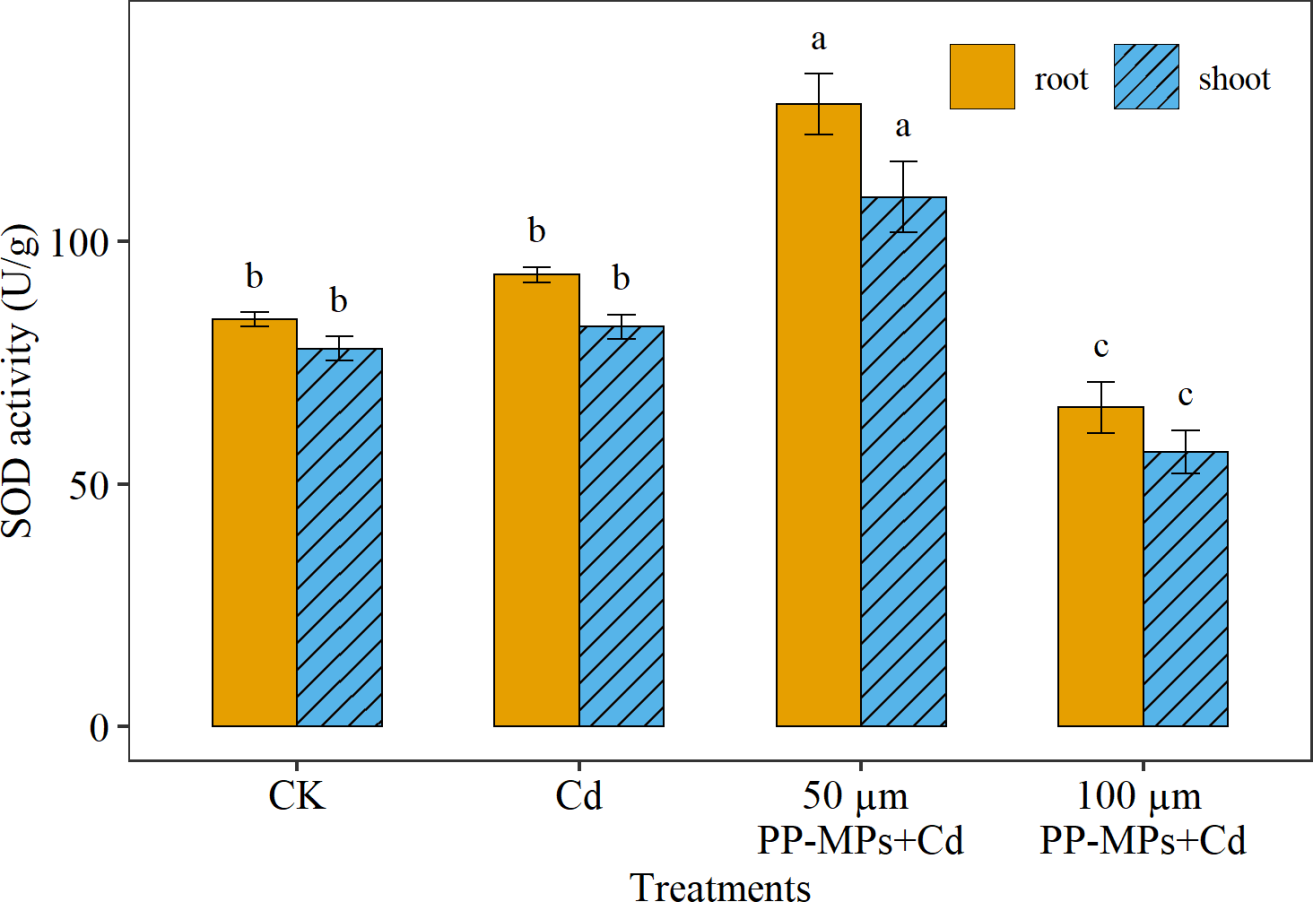
Impact of Cd (40 mg/L) and PP-MPs (50 and 100 µm) on superoxide dismutase activity (SOD) of the root and shoot on the seventh day. Values are the mean of six replicates ± standard deviation. Different alphabets represent the substantial differences between treatment groups for the root, and shoot, where CK represents the control check, Cd represents the cadmium, and PP-MPs represents the polypropylene microplastics.

Maximum SOD activity was noticed in the root (12% higher) than in the shoot. However, in contrast with POD and CAT, significantly higher SOD activity was recorded in 50 μm PP-MPs+Cd treatment followed by the Cd treatment group, CK, and 100 μm PP-MPs+Cd treatment group (**Figure 5**). The SOD activity significantly responds to PP-MPs particle size, with higher SOD activity in 50 μm PP-MPs+Cd treatment group (53% and 50% in the root and the shoot) and lower SOD activity in the 100 μm PP-MPs+Cd treatment group (21% and 27% in root and shoot) compared to CK.

## 4 Discussion

The MPs have been extensively studied over the last decade, yet little is known regarding their impact on cultivated lands (Colzi et al., 2022; Iqbal et al., 2023). Additionally, MPs polymers are hydrophobic and can leach and adsorb contaminants like aromatic hydrocarbons, polychlorinated biphenyls polycyclic organo-chlorine pesticides, and heavy metals (Li et al., 2018; Dong et al., 2020; Xin et al., 2020). In the past, the combined effect of MPs and heavy metals (particularly Cd) have rarely been explored (Kaur et al., 2022). Therefore, in this work, the impact of Cd (40 mg/L) and the combined impact of Cd with PP-MP (50 and 100 µm) has been investigated for seed germination and vigor, seedling length, seedling weight, and enzyme activities. Several seed vigor indices were measured in this study, *viz.*, GR, GI, MGT, and GE. The germination rate is a primary stage in the plant lifecycle that significantly affects crop yield (Zhang et al., 2020). However, delay in germination and seedling malformation can reduce crop production (Kim et al., 2022). In our study, higher seed GR implies that pollutants have a lesser effect on seed germination. However, with the increase in PP-MPs size, the inhibitory effect increase for seed GR. Bosker et al. (2019) reported similar results, demonstrating that MPs decrease garden cress GR, and the inhibitory effect increases with the size of the plastic. Furthermore, Pignattelli et al. (2020) demonstrated an inhibitory impact of polystyrene-MPs on *Lepidium sativum*, with a germination inhibition rate of 55%. Kaur et al. (2022) also noted the inhibitory effects of PP-MPs on rice seeds GR. Regarding GI, a higher seed GI indicates that the seed is growing in the optimum environment. In the current study, the PP-MPs combined with Cd demonstrated a significant decrease in GI on the third day compared with the seventh day wheat seedling, which can be related to aggregate development by PP-MPs over time, which might hinder seed water absorption (Kaur et al., 2022). In the case of MGT, no significant differences were found between treatments, with the exception of the 100 µm PP-MPs+Cd treatment group. Similarly, no significant results for MGT was found by Shi et al. (2022) for tomato exposed to various MPs. The GE is an important indicator of seed GR and vitality. It is expressed as the percentage of seeds that germinate within a specific time period (in this study, 3 days). Our results showed increase in GE under Cd treatment, yet it decreased under 50 µm PP-MPs+Cd and 100 µm PP-MPs+Cd treatment groups. These findings are on par with the study conducted by Bosker et al. (2019) who demonstrated that deposition of MPs on seed pores causes physical blocking and impedes water and nutrient uptake, seed germination, and seed vigor.

The current study explicitly demonstrated the deleterious impacts of Cd and PP-MPs+Cd on the GR, GI, MGT, and GE. The single Cd treatment group had a positive or slight adverse effect on the various seed germination indices. The co-existence of heavy metals such as Cd, Cu, and MPs has been shown to influence heavy metal toxicity and bioavailability (Patil et al., 2021). Phytotoxic impacts in plants can indeed be influenced by the adsorption capacity of MPs, which, in turn, is influenced by their type, shape, and size. Wang et al. (2020a) demonstrated that polystyrene MPs and high-density polyethylene adversely affect maize growth, resulting in higher phytotoxicity in conjunction with Cd. Despite the fact that Cd had no considerable influence on seed germination indices, the deleterious impacts were considerably increased under combined Cd and PP-MPs treatments.

In the case of root and shoot growth, a single application of Cd adversely affected the root and shoot growth of wheat seedlings, while the amalgamation of Cd with PP-MPs (50 µm) abated the overall toxicity caused by a single application of Cd. Therefore, it can be assumed that 50 µm PP-MPs have some detoxifying activity on Cd, which may be brought on by MPs adsorption of contaminants. This finding agrees with Gao et al. (2019) findings, which showed that the smaller the MPs, the greater their adsorption capability for various heavy metals, while the combination of Cd with 100 µm PP-MPs showed no difference. Dong et al. (2022) observed that MPs interacted with root exudates of *Oryza sativa*, leading to a reduction in iron plaque formation and subsequently inhibiting arsenic uptake in plants. In contrast, Gu et al. (2021) found a synergistic inhibitory effect on wheat root growth when exposed to Cd and polyvinyl chloride MPs. Likewise, Wang et al. (2020a) reported phytotoxic effects on maize growth caused by a combination of Cd and a high amount of high-density polyethylene. Zou et al. (2022) demonstrated that high concentrations of low-density polyethylene MPs (1.35 mg/kg), either alone or in co-occurrence with Cd, hindered the growth of *Solanum nigrum* L. instead of alleviating Cd toxicity.

Regarding the DW of wheat seedlings, the combination of Cd and PP-MPs did not significantly reduce the DW of wheat seedlings when compared to CK. Yet, the combination of Cd and 100 µm PP-MPs substantially decreases the DW of wheat seedlings on third day. However, on the seventh day, a non-significant decrease in DW was observed in the 100 µm PP-MPs+Cd treatment group, which is consistent with the findings of Kaur et al. (2022), who discovered no significant difference in DW among treatments in rice seedlings. According to our previous study by Wang et al. (2021b), small-sized polyethylene MPs had no discernable effect on the DW of *Glycine max* sprouts. However, exposure of wheat seedlings to larger-size MPs combined with Cd had a significant adverse impact on DW. The MP that reaches micro- and nanometer levels in the environment can enter the plant root, shoot, and leaves and accumulate in their tissues, resulting in the inhibition of photosynthesis, which ultimately leads to a decline in DW (Pignattelli et al., 2020). Additionally, MPs can also block the stomata of plant cell walls, hindering water absorption and nutrient transport, ultimately affecting plant growth (de Souza Machado et al., 2019; Yin et al., 2021), and can pose a potential danger to human health and development in edible plants. Although some studies show that MPs have their own effects on the environment’s chemical and physical properties, microorganism and enzyme activity, plant growth, and development, yet its toxic mechanism remains unclear (Yin et al., 2021). A study by de Souza Machado et al. (2019) observed that polystyrene MPs increased the DW of scallion roots, whereas PP-MPs decreased it. The experiment also revealed that the DW of the rhizome decreased under the treatment of polyamide MPs but increased under the treatment of polypropylene MPs.

Plants exposed to environmental stress such as MPs and heavy metals produce huge free radicals and ROS under stress (Choudhury et al., 2013). To counteract the harmful impacts of ROS, plants rely on their antioxidant enzyme system (Choudhury et al., 2013), which includes POD, CAT, and SOD. The SOD is the primary substance for removing free radicals in plants, catalyzing the transformation of superoxide anion into H_2_O_2_ and O_2_ (Lin et al., 2017). On the other hand, POD and CAT are responsible for removing H_2_O_2_ (Lin et al., 2017). These three enzymes collaborate to stabilize free radicals and prevent biochemical and physiological alterations in plants induced by free radicals (Lin et al., 2017; Sun et al., 2018; Yin et al., 2021). Consequently, measuring oxidase activity in plants can provide insight into the extent of stress that they are experiencing (Zhou et al., 2021a). The interaction between Cd and PP-MPs resulted in a higher of POD, CAT, and SOD enzymes activities in the current study. Dong et al. (2021) and Dong et al. (2022) stated that the co-occurrence of heavy metals and MPs escalates oxidative stress in plants. In general, elevated POD activity was noted in the root of wheat seedlings due to increased adsorption of Cd and PP-MPs, compared to the shoot. Furthermore, under 50 μm PP-MPs+Cd co-treatment, the toxic effect on wheat root and shoot was reduced. Increased CAT enzyme activity indicates high H_2_O_2_ production as a result of external stresses. Similar to POD, CAT activity was detected to be higher in the root than in the shoot in Cd and 100 μm PP-MPs+Cd treatment group. In contrast, Kaur et al. (2022) reported higher CAT activity in the shoot than in the root in rice seedlings exposed to Cd and PP-MPs stress. The CAT activity in the root and shoot differs between plants and is affected by the morphological/anatomical structure and chemical composition of the plant parts (Nesic et al., 2005). The higher SOD activity was detected in 50 μm PP-MPs+Cd treatment. This increase in SOD activity is thought to be an attempt by the plants to protect themselves from the damage caused by ROS, while reduced SOD activity under Cd and 100 μm PP-MPs+Cd treatment was probably due to higher ROS and antioxidizing enzymes leading to membrane damage, lipid peroxidation, and inactivation of SOD enzymes (Dong et al., 2020). In the previous study, the MPs 75–150 µm in size increased membrane instability in maize due to increased H_2_O_2_ production (Pehlivan and Gedik, 2021). In the case of plant organs, the PP-MPs may have amplified the Cd desorption and adsorption rate, and its translocation in plants, resulting in a higher uptake of Cd in roots than in shoots. However, the precise mechanisms underlying MP’s induction of oxidative stress remain unknown, as many studies have linked an increase in ROS to plant surface injuries caused by MPs abrasion (Kalčíková et al., 2017), chemical compounds leaching from absorbed MPs (Verla et al., 2019), a water-deficit condition caused by changes in soil structure (Wan et al., 2019), and a disruption in the photosynthesis process (Dong et al., 2020).

The MPs and heavy metals respond differently to various plant species based not only on their physiochemical properties but also on the plant species and the surroundings. Thus, it is necessary to conduct experiments involving various combinations of plant species, MPs, and heavy metals to understand the fate, behavior, bioavailability, and deleterious effects of MPs and toxic heavy metals in conjunction in diverse soil ecosystems.

## 5 Conclusion

Microplastics (MPs) are newly identified pollutants that have become ubiquitous in all ecosystems worldwide. Similarly, cadmium (Cd), a major heavy metal contaminant in soil, is also widely distributed globally. In the present study, we investigated the effects of Cd alone and in combination with polypropylene microplastics (PP-MPs) on wheat germination, seed vigor, root and stem growth, and oxidative stress. Our results show that the co-contamination of PP-MPs and Cd significantly affected these parameters. For example, 100 µm PP-MPs+Cd showed a 26%, 25%, and 38% decline in germination rate, germination energy, and germination index when compared to CK. In contrast, mean germination time depicted an increase of 4%. Similarly, the growth of wheat root and shoot was significantly inhibited under 100 µm PP-MPs+Cd by 8% and 10% on the third and seventh day when compared to CK. The co-contamination disrupted the regulation mechanisms of peroxidase and catalase, leading to decreased synthesis amounts compared with CK. Superoxide dismutase synthesis was inhibited at 100 µm PP-MPs+Cd by 22% and 27% in the root and shoot of wheat seedlings when compared with CK. Overall, these findings indicate that the presence of Cd and PP-MPs in agricultural soil can negatively impact plant growth and development, ultimately leading to reduced crop yield. Our research contributes to filling the knowledge gap about the toxicity of Cd and its interaction with environmental contaminants such as PP-MPs. To determine the irreversible effects of these contaminants on plant and soil ecosystems, future large-scale experiments should be conducted using a variety of MPs with different shapes, sizes, and polymer types, and other naturally occurring heavy metals.

## Data availability statement

The original contributions presented in the study are included in the article/**Supplementary Material**. Further inquiries can be directed to the corresponding author.

## Author contributions

ZH: methodology, experimentation, plant analysis, writing—original draft, and visualization. RO: conceptualization, statistical analysis, visualization writing—original draft—review and editing. YL: methodology, writing—review and editing. LW: conceptualization, methodology, writing—review and editing, and supervision. MX: conceptualization, methodology, writing—review and editing, supervision. All authors contributed to the article and approved the submitted version.
