# Supplementary material for: Analyzing the impacts of cadmium alone and in co-existence with polypropylene microplastics on wheat growth
Source: Front Plant Sci. 2023 Aug 10;14:1240472. doi: 10.3389/fpls.2023.1240472 (PMC10449543; doi:10.3389/fpls.2023.1240472)
Supplement: Supplementary file 1 [file DataSheet_1.docx]

Supplementary Material

Analyzing the impacts of cadmium alone and in coexistence with polypropylene microplastics on wheat growth

Zhiwei Han^1^, Raheel Osman^2, 3^, Yi Liu^1^, Zhangdong Wei^1^, Lin Wang^1, 2, 3*^ and Ming Xu^2,3,4*^

^1^Miami College, Henan University, Kaifeng, China

^2^College of Geography and Environmental Science, Henan University, Kaifeng, China

^3^Henan Key Laboratory of Earth System Observation and Modeling, Henan University, Kaifeng, China

^4^BNU-HKUST Laboratory for Green Innovation, Beijing Normal University, Zhuhai, China

*** Correspondence:**Corresponding Author
[wanglin@henu.edu.cn](mailto:wanglin@henu.edu.cn); [mingxu@henu.edu.cn](mailto:mingxu@henu.edu.cn)

# Supplementary Figures and Tables

## Supplementary Figures


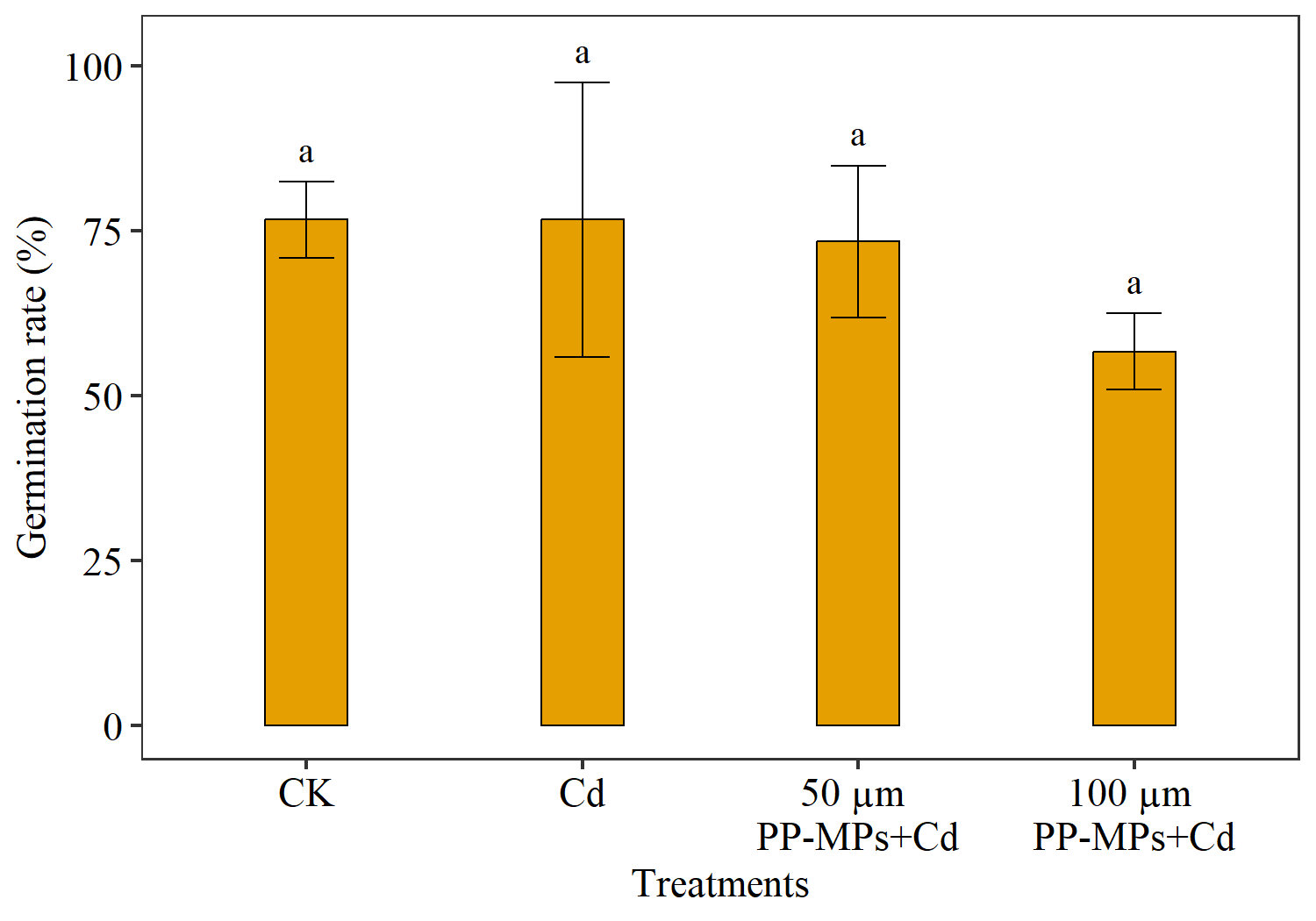


**Figure S1.** Effect of single and combined toxicity of PP-MPs and Cd on the germination rate of wheat seeds**.** Same letters indicate insignificant differences between the treatments. Where CK means control check; Cd means cadmium; PP-MPs means polypropylene microplastics.


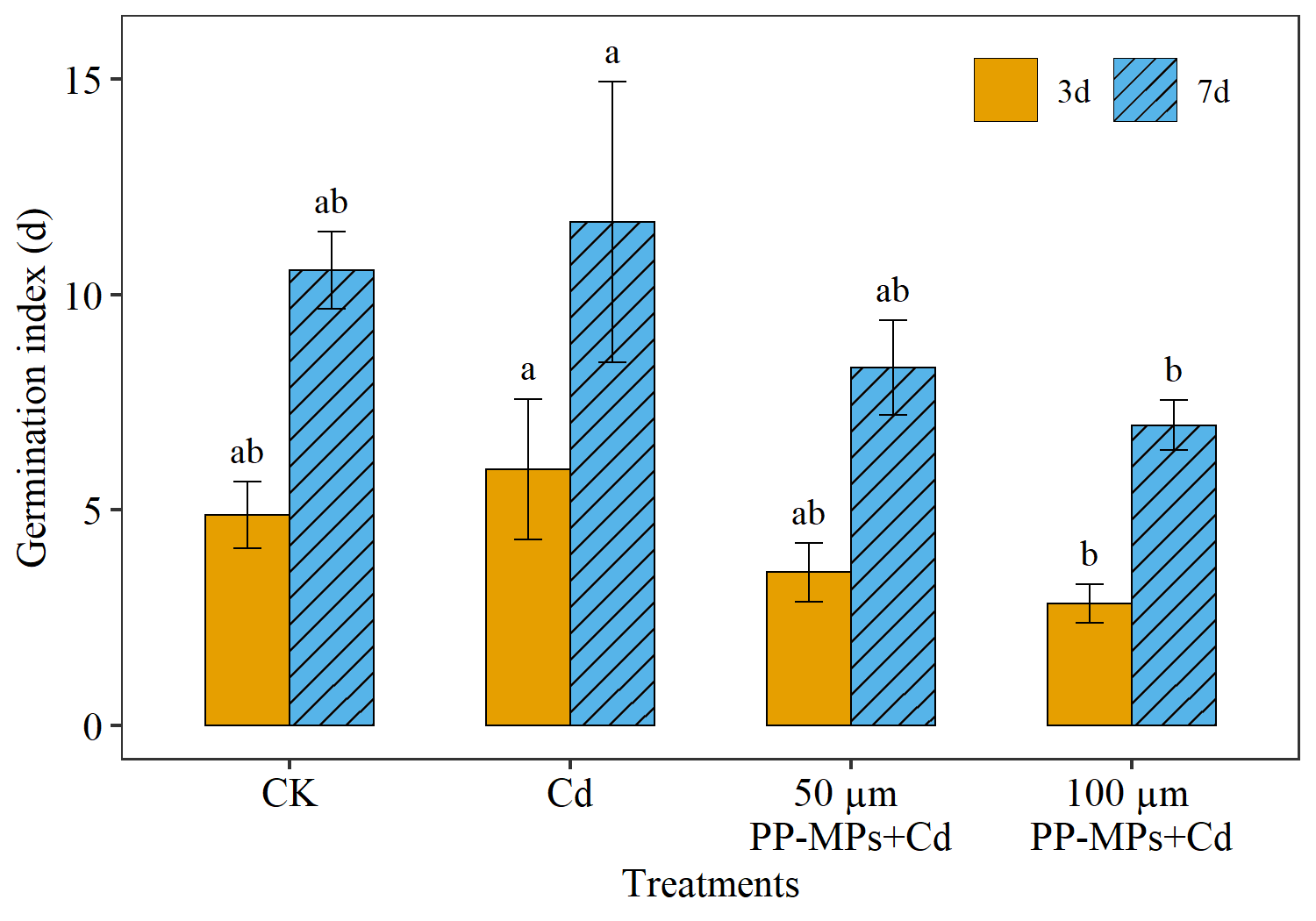


**Figure S2.** Effect of single and combined treatment of PP-MPs and Cd on 3^rd^ and 7^th^ day mean germination index of wheat seeds**.** Different alphabets represent the significant differences between various treatment groups on 3^rd^ and 7^th^ day. Where CK means control check; Cd means cadmium; PP-MPs means polypropylene microplastics.


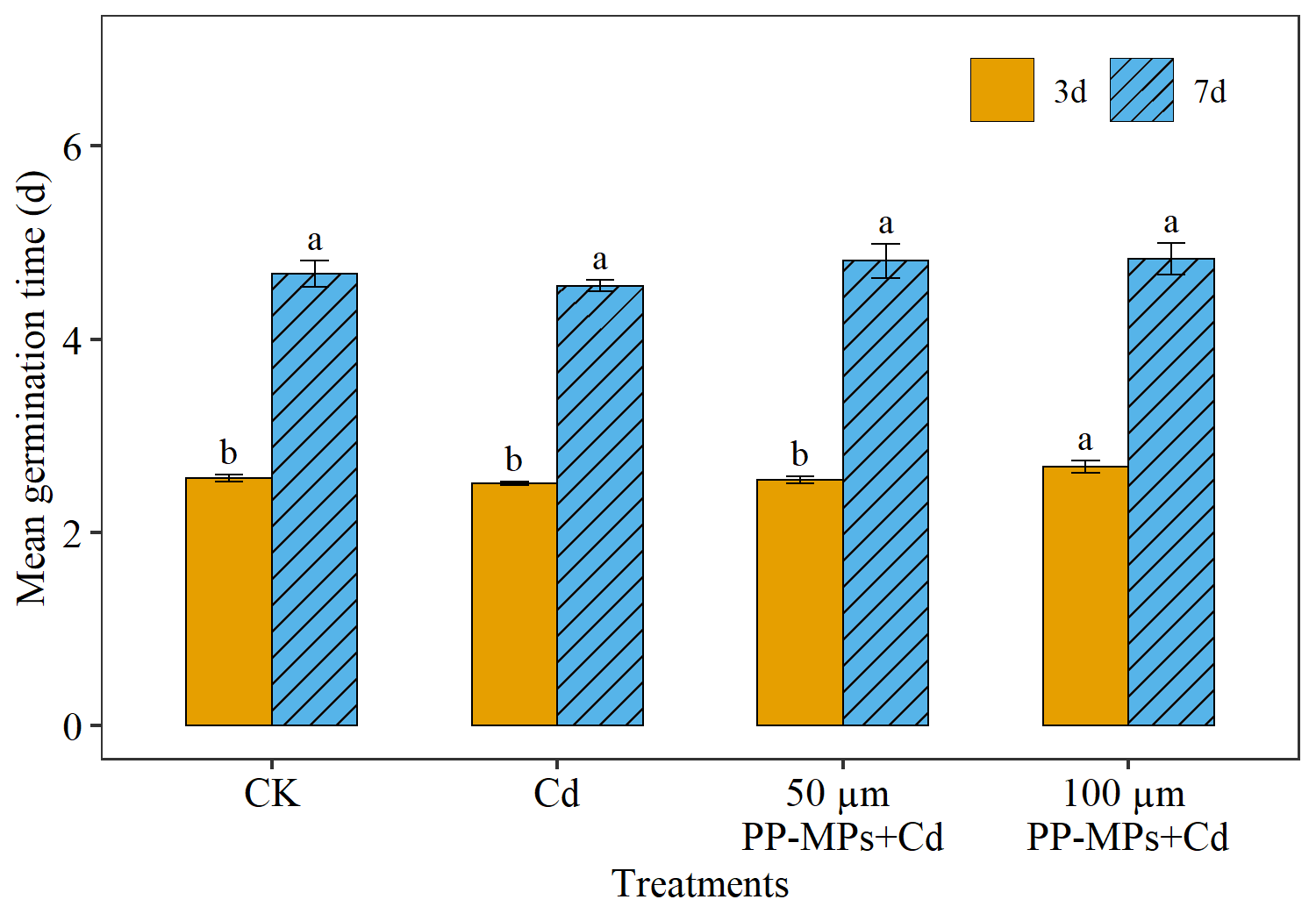


**Figure S3.** Effect of single and combined treatment of PP-MP and Cd on germination vigor of wheat seeds. Different alphabets represent the significant differences between various treatment groups on 3^rd^ and 7^th^ day. Where CK means control check; Cd means cadmium; PP-MPs means polypropylene microplastics.


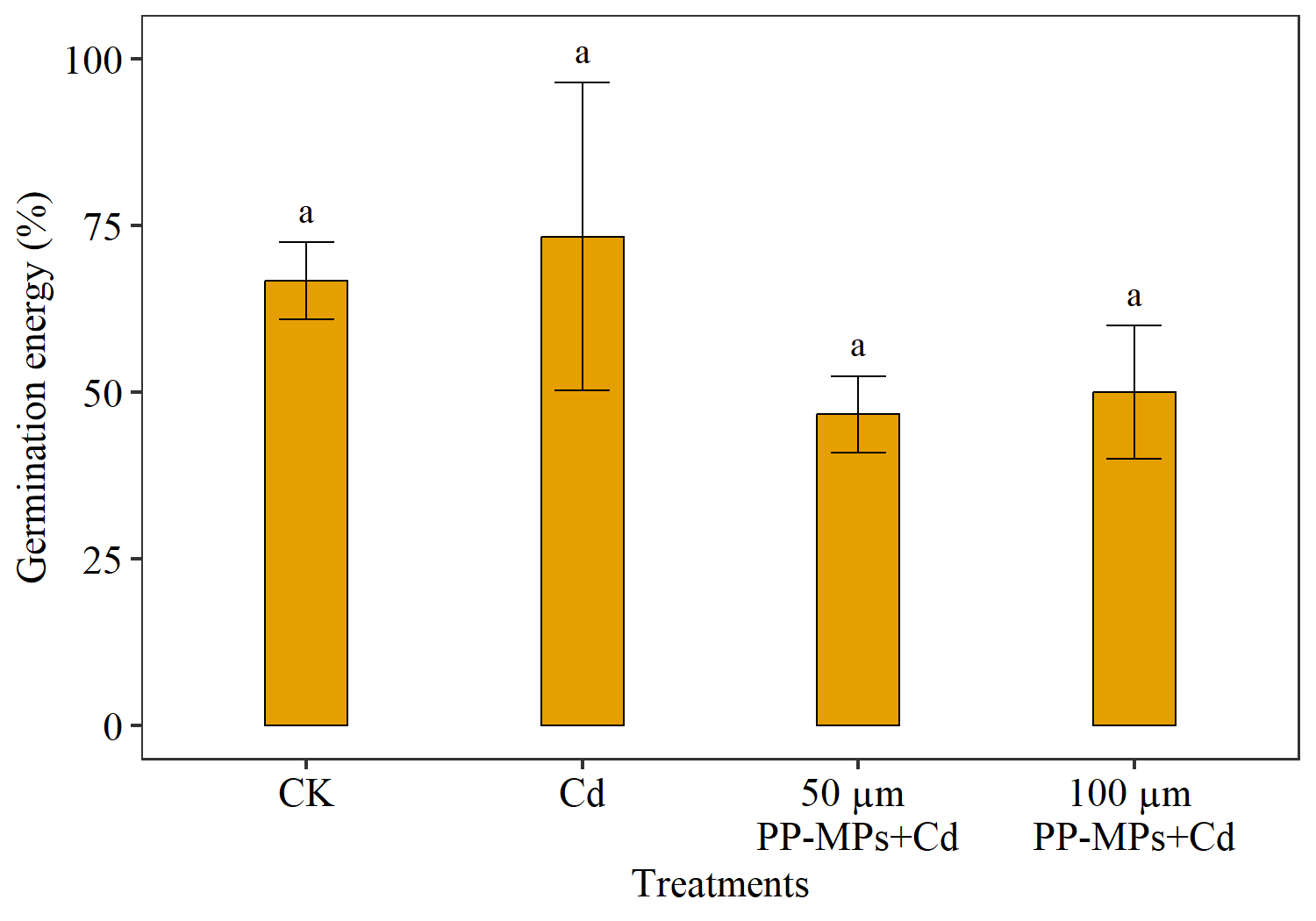


**Figure S4.** Effect of single and combined toxicity of PP-MPs and Cd on germination energy of wheat seeds. Same letters indicate insignificant differences between the treatments. Where CK means control check; Cd means cadmium; PP-MPs means polypropylene microplastics.
